# The Hsp72 and Hsp90α mRNA Responses to Hot Downhill Running Are Reduced Following a Prior Bout of Hot Downhill Running, and Occur Concurrently within Leukocytes and the Vastus Lateralis

**DOI:** 10.3389/fphys.2017.00473

**Published:** 2017-07-12

**Authors:** James A. Tuttle, Bryna C. R. Chrismas, Oliver R. Gibson, James H. Barrington, David C. Hughes, Paul C. Castle, Alan J. Metcalfe, Adrian W. Midgley, Oliver Pearce, Chindu Kabir, Faizal Rayanmarakar, Sami Al-Ali, Mark P. Lewis, Lee Taylor

**Affiliations:** ^1^Muscle Cellular and Molecular Physiology Research Group, Department of Sport Science and Physical Activity, Institute of Sport and Physical Activity Research, University of Bedfordshire Bedford, United Kingdom; ^2^Sport Science Program, College of Arts and Sciences, Qatar University Doha, Qatar; ^3^Centre for Human Performance, Exercise and Rehabilitation, Division of Sport, Health and Exercise Sciences, Department of Life Sciences, Brunel University London London, United Kingdom; ^4^Department of Neurobiology, Physiology and Behavior, University of California, Davis Davis, CA, United States; ^5^School of Exercise and Health Sciences, Edith Cowan University Perth, WA, Australia; ^6^Department of Sport and Physical Activity, Edgehill University Ormskirk, United Kingdom; ^7^Milton Keynes University Hospital Milton Keynes, United Kingdom; ^8^National Centre for Sport and Exercise Medicine, School of Sport, Exercise and Health Sciences, Loughborough University Loughborough, United Kingdom; ^9^School of Sport, Exercise and Health Sciences, Loughborough University Loughborough, United Kingdom; ^10^ASPETAR, Qatar Orthopedic and Sports Medicine Hospital Doha, Qatar

**Keywords:** downhill running, heat shock response, heat stress, heat tolerance, preconditioning, cross tolerance, thermotolerance

## Abstract

The leukocyte heat shock response (HSR) is used to determine individual's thermotolerance. The HSR and thermotolerance are enhanced following interventions such as preconditioning and/or acclimation/acclimatization. However, it is unclear whether the leukocyte HSR is an appropriate surrogate for the HSR in other tissues implicated within the pathophysiology of exertional heat illnesses (e.g., skeletal muscle), and whether an acute preconditioning strategy (e.g., downhill running) can improve subsequent thermotolerance. Physically active, non-heat acclimated participants were split into two groups to investigate the benefits of hot downhill running as preconditioning strategy. A hot preconditioning group (HPC; *n* = 6) completed two trials (HPC1_HOTDOWN_ and HPC2_HOTDOWN_) of 30 min running at lactate threshold (LT) on −10% gradient in 30°C and 50% relative humidity (RH) separated by 7 d. A temperate preconditioning group (TPC; *n* = 5) completed 30 min running at LT on a −1% gradient in 20°C and 50% (TPC1_TEMPFLAT_) and 7 d later completed 30 min running at LT on −10% gradient in 30°C and 50% RH (TPC2_HOTDOWN_). Venous blood samples and muscle biopsies (vastus lateralis; VL) were obtained before, immediately after, 3, 24, and 48 h after each trial. Leukocyte and VL Hsp72, Hsp90α, and Grp78 mRNA relative expression was determined via RT-QPCR. Attenuated leukocyte and VL Hsp72 (2.8 to 1.8 fold and 5.9 to 2.4 fold; *p* < 0.05) and Hsp90α mRNA (2.9 to 2.4 fold and 5.2 to 2.4 fold; *p* < 0.05) responses accompanied reductions (*p* < 0.05) in physiological strain [exercising rectal temperature (−0.3°C) and perceived muscle soreness (~ −14%)] during HPC2_HOTDOWN_ compared to HPC1_HOTDOWN_ (i.e., a preconditioning effect). Both VL and leukocyte Hsp72 and Hsp90α mRNA increased (*p* < 0.05) simultaneously following downhill runs and demonstrated a strong relationship (*p* < 0.01) of similar magnitudes with one another. Hot downhill running is an effective preconditioning strategy which ameliorates physiological strain, soreness and Hsp72 and Hsp90α mRNA responses to a subsequent bout. Leukocyte and VL analyses are appropriate tissues to infer the extent to which the HSR has been augmented.

## Introduction

Preconditioning of an individual using environmental stressors, with the intent of ameliorating physiological and cellular stress in extreme conditions has applications for athletic, military and occupational populations (Taylor et al., 2012; Lee et al., 2014). One pathway for preconditioning these populations is the initiation of the heat shock response (HSR) which is characterized by induction of heat shock proteins (Hung et al., 2005; Madden et al., 2008; Taylor et al., 2012). The leukocyte HSR, principally heat shock protein 72 (HSP72; protein and mRNA) is used to indicate the extent of cellular heat acclimation (Amorim et al., 2015), and identify individuals at risk of exertional heat illnesses within athletic, military and occupational settings (Moran et al., 2006; Marshall et al., 2007; Ruell et al., 2007). This is primarily due to the role of Hsp72 mRNA and HSP72 as markers of the cellular stress response and thermotolerance [attenuated cellular stress response suggests a greater likelihood of cellular survival (Kampinga et al., 1995; Theodorakis et al., 1999)] in response to isolated, combined, and cross-environmental stressors (Gibson et al., 2017). Ideally the assessment of thermotolerance would take place in skeletal muscle due to its important role in locomotion and exertional heat illness pathophysiology (Sawka et al., 2011). Unfortunately, obtaining multiple muscle biopsies prior to relocation to a hot environment is not always viable for ethical, performance, cost, comfort and medical reasons (MacInnis et al., 2017). Leukocytes are a desirable tissue site for determining thermotolerance given the relative ease by which they can be collected, and because leukocytes, as circulating cells, are exposed to both systemic signals and to signals of the perfused tissues (Sonna et al., 2007). As such Hsp72 mRNA from leukocytes has been utilized as a surrogate to skeletal muscle samples with inferences made from changes in circulating intracellular sites across many exercise, heat, and nutritional experiments whereby the cellular stress response and thermotolerance are augmented (Fehrenbach et al., 2000a,b, 2001; Niess et al., 2002; Connolly et al., 2004; Marshall et al., 2007; Selkirk et al., 2009; Gibson et al., 2015a,c; Tuttle et al., 2015; Mee et al., 2016). Consequently, determining whether the HSR occurs concurrently within both tissues (leukocytes and the vastus lateralis; VL) following an acute stressor (initial experimental trial), and whether this response is attenuated in both tissues following a second trial (i.e., following preconditioning), requires elucidation to assess the viability of the leukocyte HSR to represent the skeletal muscle HSR.

The Hsp72 mRNA response is particularly pertinent during this acute stress response because HSP72 protein concentrations (due to translational inhibition) may not necessary directly represent the magnitude of the cellular stress response, particularly during the early stages of adaptation to stress (Paulsen et al., 2007) and within heat intolerant individuals (Moran et al., 2006). The differential kinetics of the Hsp72 response in the VL [typically delayed, peak between 24 h and 7d; (Morton et al., 2006; Tupling et al., 2007)] compared to leukocyte subsets [0–24 h (Fehrenbach et al., 2000a; Oehler et al., 2001)] suggests the leukocyte Hsp72 mRNA specific response which peaks within 0–3 h (Fehrenbach and Northoff, 2001; Neubauer et al., 2014), is more practical (shorter sampling time course required) for assessing the cellular stress response in the VL for comparative purposes. In addition to Hsp72 mRNA, Hsp90α mRNA is of interest due to its important role within restoration of proteostasis (Kourtis and Tavernarakis, 2011; van Oosten-Hawle et al., 2013), regulation of the transmission of signaling cascades (Taipale et al., 2010), recovery of global protein synthesis (Duncan, 2005) and regulation of cellular repair (Erlejman et al., 2014). Additionally it is unknown if the physiological signals e.g., increases in systemic temperature (Gibson et al., 2016), which elicit increases in leukocyte Hsp72 and Hsp90α mRNA transcription to damaging (Tuttle et al., 2015), and non-damaging exercise-heat stress (Gibson et al., 2015c), are as relevant in skeletal muscle. The current study also sought to investigate the gene transcript response of another HSP, glucose regulated protein 78 mRNA (Grp78 mRNA) given its ability to indicate when the unfolded protein response ends (Ron and Walter, 2007). Importantly Grp78 mRNA may also act as a biomarker of thermotolerance within heat intolerant individuals where Heat Shock factor-1 (HSF-1) signaling and Hsp72 and Hsp90α mRNA transcription are attenuated (McMillan et al., 1998). However, it is currently unclear if previous *in vitro* observations demonstrating the role of Hsp72 and Hsp90α mRNA in the cellular stress response (Heldens et al., 2011) occur within human leukocytes and skeletal muscle (VL) *in vivo* (i.e., following exercise, and exercise and heat related stressors).

Experimental aims were to determine whether a prior bout of hot downhill running [eliciting large changes in exercising rectal temperature (T_re_) and delayed onset muscle soreness (DOMS)], when compared to a temperate flat run, could provide a preconditioning effect relative to attenuation of the VL Hsp responses (Hsp72, Hsp90α, and Grp78 mRNA) during a subsequent trial of hot downhill running 7 d later. The second experimental aim was to determine whether this response occurred concurrently within leukocytes and the VL. It was hypothesized that a prior bout of hot downhill running would attenuate both the VL and leukocyte Hsp72 and Hsp90 mRNA responses during a second trial, and that a significant relationship between the VL and leukocyte Hsp72 and Hsp90 mRNA responses following the first trial would exist.

## Methods

### Ethical approval

The protocol was approved by the University of Bedfordshire's Sport Science and Physical Activity Departmental Human Ethics Committee and all participants signed informed consent in accordance with the ethical standards outlined in the 1964 Declaration of Helsinki.

### Participants

Demographic variables were recorded for 11 male Caucasian participants (see Table 1) who were non-smokers and were not heat acclimated (experimental trials completed between January and March, within the UK; average temperatures 1.5°C–8.1°C). Body mass (kg) and height (cm) were measured with a single set of mechanical scales (Weylux Marsden 424 London, UK) and a stadiometer (Harpenden HAR- 98.602, Crymych, UK) respectively. Body composition was measured using air displacement plethysmology (Bod Pod 2000A, Cranlea, UK). The lactate threshold (LT) and maximum oxygen uptake ($\dot{\text{V}}$O_2max_) were determined using a graded treadmill test (Winter et al., 2007). This test consisted of 6–8 incremental 3 min stages at a 1% gradient. Participants started running at 8–9 km.h^−1^ and running velocity was increased by 1 km.h^−1^ per stage until exhaustion. Fingertip capillary blood samples (40 μL) were taken at rest and the end of each 3 min stage to determine blood lactate concentrations (B[La]). Blood lactate concentrations were plotted against running velocity to determine LT which was defined as the first sustained B[La] increase above baseline. Pulmonary gas exchange was measured breath by breath using an online gas analysis system (Cortex Metalyser 3B, Biophysik, Leipzig, Germany) to determine changes in oxygen uptake ($\dot{V}$O_2_) with the highest $\dot{V}$O_2_ attained over a 30 s period accepted as $\dot{V}$O_2max_.

**Table 1:** Participant demographic characteristics.

	**Temperate preconditioning group (TPC; *n* = 5)**	**Heat preconditioning group (HPC; *n* = 6)**	**Group sig (*p* < 0.05)**
Age (Years)	20.4 ± 2.8	21.7 ± 2.3	0.426
Height (cm)	177 ± 7	180 ± 10	0.593
Body Weight (kg)	75.2 ± 18.1	76.1 ± 12.3	0.931
	50.8 ± 6.9	52.8 ± 5.0	0.587
% Lean mass	88.3 ± 11.5	86.8 ± 4.8	0.777
% Body Fat	11.7 ± 11.5	13.2 ± 4.8	0.777

*Values are expressed as mean ± SD. mL.kg.min^−1^ (milliliters per kilogram per minute), $\dot{V}$O_2max_ (maximum oxygen uptake)*.

Sample size calculations of Hsp72 mRNA were determined via G.Power 3.1, (Universität Dusseldorf, Germany; Faul et al., 2009) using data from a previous paper (Mestre-Alfaro et al., 2012). For a two tailed test with an alpha of 0.05 and power of 0.8, Six participants were required to find an Hsp72 mRNA increase of 3.8-fold significant. This sample size is ≥ others in the field (Puntschart et al., 1996; Febbraio and Koukoulas, 2000; Fehrenbach and Northoff, 2001; Fehrenbach et al., 2003; Liu et al., 2004; Mee et al., 2016).

### Experimental design

**Figure 1:**
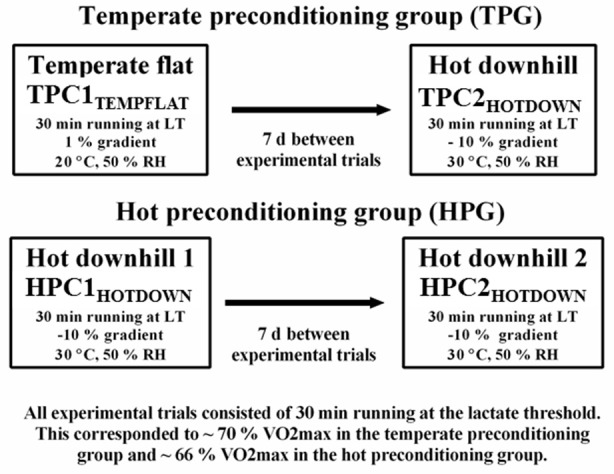
Schematic representation of the experimental design. $\dot{V}$O_2max_ (maximum oxygen uptake).

Participants were split into two experimental groups (see Figure 1). The temperate (TPC; five participants) and HOT (HPC; six participants) preconditioning groups (conditions) both featured two exercise trials separated by 7 d:

*TPC Exercise trial (1):* Temperate flat (TPC1_TEMPFLAT_) which involved 30 min running at the LT on a 1% gradient in 20°C, 50% RH. *TPC Exercise trial (2):* 7 d post TPC1_TEMPFLAT_, hot downhill (TPC2_HOTDOWN_) which involved 30 min downhill running at the LT on a −10% gradient in 30°C, 50% RH.

*HPC Exercise trial (1):* Hot downhill (HPC1_HOTDOWN_) which involved 30 min downhill running at the LT on a −10% gradient in 30°C, 50% RH. *HPC Exercise trial (2):* 7 d post HPC1_HOTDOWN_, hot downhill 2 (HPC2_HOTDOWN_) which involved 30 min downhill running at the LT on a −10% gradient in 30°C, 50% RH.

Previous work from our research group has demonstrated that the leukocyte Hsp72 and Hsp90α mRNA responses are larger following exercise in hot compared to temperate environments (Gibson et al., 2015c, 2016), and following downhill compared to flat running (Tuttle et al., 2015). It is known that downhill running is an effective whole body preconditioning strategy (Shima et al., 2008; Touchberry et al., 2012; Isanejad et al., 2015), consequently, an acute preconditioning trial featuring both stressors (hot environmental conditions and downhill running; hot downhill running) was selected in the current experimental design to maximize stimuli to initiate the HSR and subsequent cellular preconditioning. This was compared to a temperate flat trial (flat running in a temperate environment) where no change in leukocyte Hsp72 and Hsp90α mRNA has been previously observed (Tuttle et al., 2015) and thus no preconditioning effect was hypothesized to occur. A 7 d period between trials was selected to ensure any spontaneous preconditioning effect from exercise stress on core temperature (Barnett and Maughan, 1993) and leukocyte HSP72 (Fehrenbach et al., 2001; Lee et al., 2014), had returned to baseline following TPC1_TEMPFLAT_.

All experimental trials were completed at the running velocity which elicited the LT to minimize differences in metabolic strain between experimental trials (Baldwin et al., 2000). However, environmental temperature mediated differences still remained as relative exercise intensity is higher at the same velocity during exercise in hot environments (Lorenzo et al., 2011). All experimental trials were completed at the same time of day to minimize the influence of diurnal and circadian variations on exercise performance (Drust et al., 2005). Confounding variables were controlled for via abstinence prior to testing and throughout the testing period (see brackets for duration). These confounding variables were caffeine and alcohol (72 h), non-steroidal anti-inflammatory medications [48 h (Nielsen and Webster, 1987; Van Wijck et al., 2012)], dietary supplementation (vitamins, ergogenic aids; 30 d), exercise [7 d (Morton et al., 2006)], thermal stressors [3 months (Gibson et al., 2014)] and hypoxic and hyperbaric stressors [3 months (Taylor et al., 2010a, 2011, 2012)]. A questionnaire was administered prior to each experimental trial to determine adherence to the aforementioned experimental control measures with apparent adherence 100% in all participants.

Participants were instructed to drink 500 mL of water 2 h before each experimental trial as per the ACSM position stand (Sawka et al., 2007). Hydration status was assessed via urine osmolality (UOsm) using a handheld digital refractometer (Osmocheck, Vitech Scientific Ltd, Horsham, UK) before any pre exercise measures were obtained and immediately after exercise. All participants were euhydrated [UOsm was <600 mOsmols.kg.H_2_0 (Hillman et al., 2011, 2013)] prior to all experimental conditions and remained euhydrated during each experimental trial despite UOsm increasing (Time; *F* = 63.7, *p* < 0.001) immediately post exercise compared to basal.

## Molecular physiology measures

### Blood sampling and leukocyte isolation

Venous blood was obtained from the antecubital vein into a 6 mL EDTA tube immediately before (basal), immediately post, 3 h post, 24 h post, and 48 h post exercise. Using an adaptation of a previously validated method (Taylor et al., 2010b), 500 μL of venous blood was pipetted into 10 mL of 1 in 10 red blood cell lysis solution (10X Red Blood Cell Lysis Solution, Miltenyi Biotech, UK). Samples were incubated for 15 min at room temperature and then isolated via centrifugation at 400G for 5 min and washed twice in 2 mL phosphate-buffered saline (PBS) at 400 G for 5 min. The pellet was suspended in 1 mL of PBS, pipetted into a 1.5 mL RNase free microtube and then centrifuged at 17 000 G for 5 min at 4°C. The remaining supernatant was aspirated prior to the pellet being completely re-suspended in 200 μL of TRIzol reagent (Sigma Aldrich, Dorset, UK) and stored at −80°C for subsequent RNA extraction.

### Muscle biopsies

All biopsies were taken by medically qualified Orthopedic Surgeons, with full UK General Medical Council registration. Muscle Biopsies were obtained using a previously validated and HSP specific *in vivo* technique (Morton et al., 2006, 2007, 2008, 2009) applied to the lateral portion of the vastus lateralis. Biopsies were taken 3 cm apart in a proximal to distal fashion, under local anesthetic (2% lidocaine hydrochloride). The fascia of the muscle was specifically avoided (Trappe et al., 2013). Disposable manually primed biopsy needle guns were utilized (12 × 16, Disposable Monopty Core Biopsy Instrument, Bard Biopsy Systems, USA). Samples collected (20–30 mg) were immediately frozen in liquid nitrogen (−196°C) and stored at −80°C for later analysis. Serial biopsies were separated by 3 cm to ensure muscle damage from previous incisions did not influence the Hsp72, Hsp90α, and Grp78 mRNA responses (Khassaf et al., 2001).

Biopsy samples were later ground under liquid nitrogen to remove surrounding tissue (i.e., adipose, and connective tissue) prior to homogenization with a sonicator (T10 Basic, IKA, Thermo Fisher Scientific, Loughborough, UK) on ice in 1 mL TRIzol reagent followed by a 10 min incubation period on ice, in preparation for RNA extraction.

### RNA extraction

The TRIzol method was used to extract RNA from the biopsy samples and the leukocytes in accordance with manufacturer instructions (Invitrogen, Life Technologies, Carlsbad, USA). Quantity was determined at an optical density of 260 nm while quality was determined via the 260/280 and 260/230 ratios using a nanodrop spectrophotometer (Nanodrop 2000c, Thermo Scientific). Only samples with a 260:280 ratio of between 1.9 and 2.15 were carried forward for reverse transcription and PCR amplification detailed below.

### One step reverse transcription quantitative polymerase chain reaction (RT-QPCR)

Primers (see Table 2) were designed using primer design software (Primer Quest and Oligoanalyzer—Integrated DNA technologies). During primer design sequence homology searches were performed against the Genbank database to ensure the primers matched the gene of interest. Primers were designed to span exon-intron boundaries and avoided three or more GC bases within the last 5 bases at the 3′ end of primer to avoid non-specific binding. Further searches were performed to ensure primers did not contain secondary structures and inter or intra molecular interactions (hairpins, self-dimer and cross dimers), which can inhibit product amplification. Hsp72, Hsp90α and Grp78 relative mRNA expression was then quantified using RT-QPCR. 20 μL reactions containing 10 μL SYBR-Green RT-PCR Mastermix (Quantifast SYBRgreen Kit, Qiagen, Manchester, UK), 0.15 μL forward primer, 0.15 μL reverse primer, 0.2 μL reverse transcription mix (Quantifast RT Mix, Qiagen) and 9.5 μL sample (70 ng RNA/ μL) were prepared using the Qiagility automated pipetting system (Qiagen). Each reaction was amplified in a thermal cycler (Rotorgene Q, Qiagen) and involved reverse transcription lasting 10 min at 50°C and a transcriptase inactivation and initial denaturation phase lasting 5 min at 95°C. The PCR reaction then followed with a denaturation step lasting 10 s at 95°C and a primer annealing and extension stage lasting 30 s at 60°C repeated for 40 cycles. Fluorescence was measured following each cycle as a result of the incorporation of SYBR green dye into the amplified PCR product. Melt curves (50 to 95°C; Ramp protocol 5 s stages) were analyzed for each reaction to ensure only the single gene of interest was amplified.

**Table 2:** Primer sequences.

**Gene**	**NCBI Accession No**.	**Primer**	**Sequence (5′ → 3′)**	**Amplicon length**
β2-Microglobulin (β2-M)	NM_004048	Forward	CCGTGTGAACCATGTGACT	91
		Reverse	TGCGGCATCTTCAAACCT	
Grp78	NM_005347	Forward	TGGAGGTGGGCAAACAAAGACA	154
		Reverse	TGCTTGGCGTTGGGCATCATTA	
Hsp72	NM_005345	Forward	CGCAACGTGCTCATCTTTGA	198
		Reverse	TCGCTTGTTCTGGCTGATGT	
Hsp90α (variant 1 & variant 2)	NM_001017963 & NM_005348	Forward	AAACTGCGCTCCTGTCTTCT	180
		Reverse	TGCGTGATGTGTCGTCATCT	

*3′ (3 primer end), 5′ (5 primer end), Grp78 (Glucose regulated protein 78), Hsp72 (Heat shock protein 72), Hsp90α (Heat shock protein 90 α)*.

The relative quantification of mRNA expression for each sample (Hsp72, Hsp90α, and Grp78) was assessed by determining the ratio between the cycling threshold (C_T_) value of the target mRNA and the C_T_ values for β2-Microglobulin (β2-M) mRNA. Fold change in relative mRNA expression was calculated using the 2-ΔΔC_T_ method (Schmittgen and Livak, 2008). β2-Microglobulin was used as a housekeeping gene as it was stable between experimental trials and across time in both the VL and leukocytes, as previously observed following exercise (Mahoney et al., 2004, 2008; Tuttle et al., 2015). The coefficient of variation for β2-M mRNA, Hsp72 mRNA, Hsp90α mRNA and Grp78 mRNA were 0.55, 0.34, and 0.28% respectively.

### Statistical analysis

Central tendency and dispersion are reported as the mean and standard deviation for normally distributed data and as the median and interquartile range for non-normally distributed data. Inferential statistical analyses were completed using linear mixed models for repeated measures (IBM SPSS Statistics 19, Chicago, IL) with comparisons made for main effects, two way interactions (experimental trial × time) and three way interactions (group × experimental trial × time). The best fitting covariance structure was selected by minimizing the Hurvich and Tsai's criterion (Field, 2013). Changes in Hsp72, Hsp90α, and Grp78 mRNA are presented as fold change from basal in accordance with previous literature (Tuttle et al., 2015; Gibson et al., 2015c). Where significant F ratios for main and interaction effects occurred, *post-hoc* pairwise comparisons were made with Bonferroni adjusted *p*-values. Pearson's product correlation was performed between leukocyte and vastus lateralis Hsp72 mRNA and Hsp90α mRNA before, immediately after and 3 h after TPC1_TEMPFLAT_ and HPC1_HOTDOWN._ Pearson's product correlations were also performed between physiological variables T_re_ and HR, and leukocyte and VL Hsp72 mRNA and Hsp90α mRNA immediately and 3 h after the corresponding TPC1_TEMPFLAT_ and HPC1_HOTDOWN_. The mRNA responses to TPC2_HOTDOWN_ and HPC2_HOTDOWN_ were not included in the correlational analyses given the likelihood of the prior trials to be a confounding factor due to the hypothesized preconditioning effect i.e., increase gene transcription and therefore signal post translational events to increase basal HSP (Tuttle et al., 2015). Statistical significance was accepted at *p* < 0.05 (two tailed).

## Results

### Thermoregulatory response

**Figure 2:**
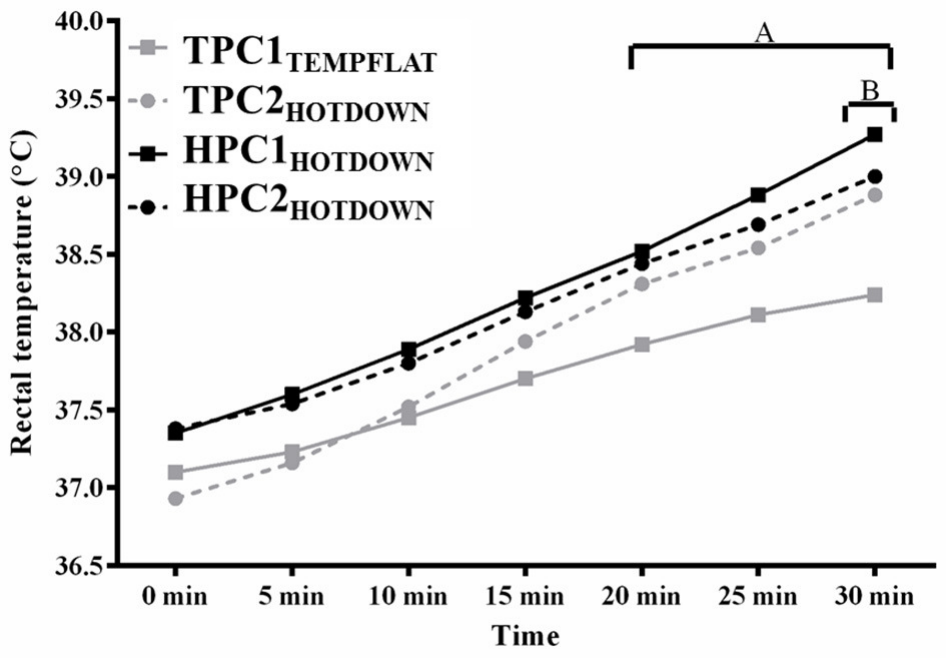
Rectal temperature (T_re_) at 0–30 min of exercise. A, T_re_ increased (*p* < 0.005) during TPC2_HOTDOWN_ compared to TPC1_TEMPFLAT_. B, T_re_ decreased (*p* = 0.017) during HPC2_HOTDOWN_ compared to HPC1_HOTDOWN_. Mean data presented. Error bars omitted to maintain clarity.

Exercising T_re_ (Figure 2) increased as main effect between 5 and 30 min (*p* < 0.001) compared to basal. Average exercising T_re_ was higher during the hot downhill running trials (HPC1_HOTDOWN_; 38.3°C; *F* = 14.3, *p* = 0.002, and TPC2_HOTDOWN_ (37.9°C; *F* = 6.1, *p* = 0.017) compared to the temperate flat trial (TPC1_TEMPFLAT_; 37.7°C). Exercising T_re_ was greater during the hot downhill trials (TPC2_HOTDOWN_; 20–30 min, *p* < 0.05, HPC1_HOTDOWN_; 5–30 min, *p* < 0.05) compared to the temperate flat trial (TPC1_TEMPFLAT_). Exercising T_re_ was also 0.3°C higher (39.3 ± 0.3°C compared to 39.0 ± 0.4°C) at 30 min during HPC1_HOTDOWN_ compared to HPC2_HOTDOWN_ (*F* = 6.1, *p* = 0.017).

**Figure 3:**
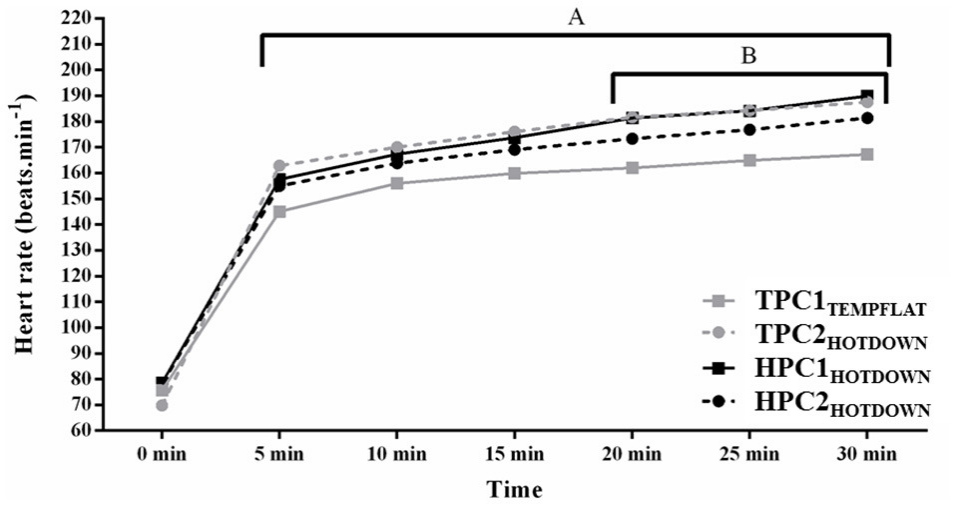
Heart rate (HR) at 0–30 min of exercise. A, increased (*p* < 0.010) during TPC2_HOTDOWN_ compared to TPC1_TEMPFLAT_ at 5–30 min. B, increased (*p* < 0.050) during HPC1_HOTDOWN_ increased compared to TPC1_TEMPFLAT_ at 20–30 min. Mean data presented. Error bars omitted to maintain clarity.

Heart rate (Figure 3) was increased compared to basal between 5 and 30 min (*p* < 0.001). Average HR was higher during TPC2_HOTDOWN_ (162 beats.min^−1^) compared to TPC1_TEMPFLAT_ (147 beats.min^−1^; *F* = 22.3, *p* = 0.001). No difference in average HR was observed between HPC1_HOTDOWN_ (161 beats.min^−1^) and TPC1_TEMPFLAT_ (*F* = 3.3, *P* = 0.096) or HPC2_HOTDOWN_ (157 beats.min^−1^; *F* = 2.8, *p* = 0.128). Heart rate was higher during the hot downhill trials (TPC2_HOTDOWN_; 5–30 min, *p* < 0.05 and HPC1_HOTDOWN_; 20–30 min, *p* < 0.05) compared to the temperate flat trial (TPC1_TEMPFLAT_). A trend for HR to be reduced during HPC2_HOTDOWN_ compared to HPC1_HOTDOWN_ between 20 and 30 min (8 beats.min^−1^; ~ *F* = 3.8, *p* = ~ 0.069) was observed.

**Figure 4:**
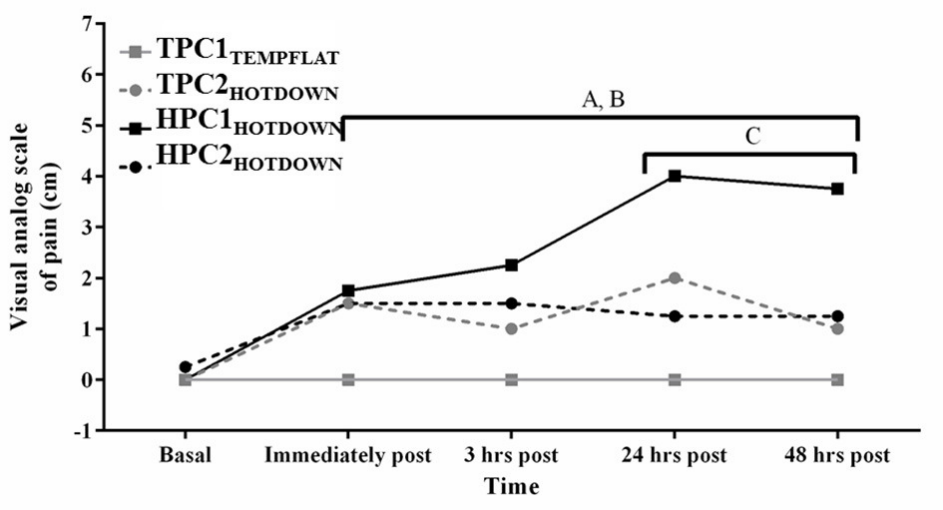
Perceived muscle soreness measured via the Visual analog scale of pain (VAS) immediately before, immediately post, 3 h post, 24 h post, and 48 h post exercise. A, increased (*p* < 0.005) during TPCHEAT_DOWN_ compared to TPC1_TEMPFLAT_. B, increased (*p* < 0.001) during HPC1_HOTDOWN_ compared to TPC1_TEMPFLAT_. C, decreased (*p* < 0.001) during HPC2_HOTDOWN_ compared to HPC1_HOTDOWN_. Median data presented. Error bars omitted to maintain clarity.

Perceived muscle soreness (indicated by the VAS; Figure 4) was increased over time as a main effect immediately post to 48 h post exercise compared to basal (*p* < 0.001). Perceived muscle soreness also increased from basal between immediately post to 48 h post exercise following TPC2_HOTDOWN_ and HPC1_HOTDOWN_ (*p* < 0.001) and between immediately post—3 h post HPC2_HOTDOWN_ (*p* < 0.05). Perceived muscle soreness was greater following the hot downhill running trials (TPC2_HOTDOWN_ and HPC1_HOTDOWN_) compared to the temperate flat running trial (TPC1_TEMPFLAT_) immediately post, (*F* = 7.2, *p* = 0.011 and *F* = 11.8, *p* = 0.002), 3 h post (*F* = 6.1, *p* = 0.019 and *F* = 9.1, *p* = 0.005), 24 h post (*F* = 12.2, *p* = 0.001 and *F* = 25.0, *p* < 0.001) and 48 h post exercise (*F* = 14.3, *p* = 0.001 and *F* = 30.4, *p* < 0.001) respectively. Perceived muscle soreness was attenuated 24 and 48 h after the second hot downhill running trial (HPC2_HOTDOWN_) compared to the first hot downhill trial (HPC1_HOTDOWN_; *F* = 12.6, *p* = 0.001 and *F* = 11.3, *p* = 0.002, respectively) in the HPC.

Quadriceps tenderness (QT; Table 3) was increased as a main effect immediately post to 48 h post exercise (*p* < 0.05) compared to basal. No difference in QT was observed between experimental trials (*P* > 0.05).

Table 3Physiological and perceptual responses.

**Table:** 

		**TPC1_TEMPFLAT_**	**TPC2_HOTDOWN_**	**HPC1_HOTDOWN_**	**HPC2_HOTDOWN_**
B[La] (mmol.l^−1^)	Basal	1.0 ± 0.3^A^	0.8 ± 0.1	0.6 ± 0.1	0.8 ± 0.2
	Immediately post	1.0 ± 0.3	1.8 ± 0.5^*^	1.6 ± 0.8^*^	1.1 ± 0.6^*^
Urine Osmolality (mOsmols.kg H_2_0)	Basal	250.0 ± 200.0	150.0 ± 40.0	165.0 ± 152.5	170.0 ± 185.0
	Immediately post	310.0 ± 60.0^B^	330.0 ± 100.0^B^	390.0 ± 267.5^B^	430.0 ± 305.0^B^
Quadriceps tenderness (% decrease in force required to elicit tenderness)	Basal	100 ± 0.0	98.9 ± 10.9	100 ± 0.0	101.4 ± 8.7
	Immediately post	87.8 ± 9.3^*^	79.3 ± 16.7^*^	85.3 ± 12.5^*^	87.0 ± 11.3^*^
	3 hrs post	86.6 ± 15.6^*^	82.3 ± 14.7^*^	88.6 ± 7.7^*^	92.3 ± 8.8^*^
	24 hrs post	88.9 ± 13.8^*^	79.9 ± 22.5^*^	73.1 ± 9.7^*^	85.3 ± 4.8^*^
	48 hrs post	93.6 ± 7.9^*^	89.0 ± 27.9^*^	82.8 ± 16.9^*^	99.3 ± 10.5^*^

**Table:** 

$\dot{V}$**O**_2_ **(ml.kg.min**^−1^**)**	**0 min**	**5 min**	**10 min**	**15 min**	**20 min**	**25 min**	**30 min**
TPC1_TEMPFLAT_	6.4 ± 1.8	34.3 ± 4.2^*^	35.1 ± 5.6^*^	36.2 ± 6.4^*^	36.1 ± 5.6^*^	35.9 ± 5.0^*^	36.0 ± 4.0^*^
TPC2_HOTDOWN_	6.0 ± 1.0	34.2 ± 6.2^*^	35.1 ± 6.5^*^	36.1 ± 7.8^*^	36.7 ± 7.8^*^	36.1 ± 6.8^*^	36.2 ± 7.2^*^
HPC1_HOTDOWN_	6.3 ± 1.5	32.0 ± 1.8^*^	32.6 ± 2.1^*^	34.4 ± 1.7^*^	35.6 ± 1.4^*^	36.7 ± 1.8^*^	37.2 ± 1.7^*^
HPC2_HOTDOWN_	6.5 ± 0.8	32.0 ± 1.3^*^	33.4 ± 1.6^*^	33.6 ± 2.5^*^	35.4 ± 2.4^*^	36.0 ± 2.7^*^	36.4 ± 2.8^*^
**RPE (Units)**							
TPC1_TEMPFLAT_	6.0 ± 0.0	10.0 ± 2.0	10.4 ± 0.8	11.8 ± 1.5	12.6 ± 1.5	13.2 ± 1.6	13.4 ± 1.6
TPC2_HOTDOWN_	6.0 ± 0.0	11.4 ± 1.3^C^	13.4 ± 0.9^C^	14.4 ± 0.6^C^	15.4 ± 0.9^C^	16.0 ± 0.8^C^	17.3 ± 1.0^C^
HPC1_HOTDOWN_	6.0 ± 0.0	11.5 ± 1.1^C^	13.2 ± 0.8^C^	14.2 ± 1.1^C^	15.3 ± 0.5^C^	16.5 ± 0.6^C^	17.5 ± 1.2^C^
HPC2_HOTDOWN_	6.0 ± 0.0	11.8 ± 0.8	12.8 ± 0.8	13.7 ± 1.0	15.1 ± 1.0	16.0 ± 1.3	17.0 ± 1.3
**TS (Units)**							
TPC1_TEMPFLAT_	4.0 ± 0.0	3.6 ± 1.1	4.4 ± 1.0	5.0 ± 0.9	5.4 ± 0.7	5.5 ± 0.6	5.5 ± 0.7
TPC2_HOTDOWN_	4.0 ± 0.0	5.1 ± 0.7^C^	5.6 ± 0.7^C^	6.0 ± 0.7^C^	6.6 ± 0.7^C^	7.0 ± 0.4^C^	7.3 ± 0.3^C^
HPC1_HOTDOWN_	4.0 ± 0.0	4.7 ± 0.5^C^	5.3 ± 0.4^C^	5.8 ± 0.5^C^	6.3 ± 0.4^C^	6.8 ± 0.4^C^	6.9 ± 0.4^C^
HPC2_HOTDOWN_	4.0 ± 0.0	4.8 ± 0.4	5.5 ± 0.7	5.7 ± 0.7	6.3 ± 0.4	6.7 ± 0.4	6.8 ± 0.4

*Values are expressed as mean ± SD for quadriceps tenderness and $\dot{V}$O_2_. Values are expressed as median ± IQR for B[La], RPE, TS and Urine Osmolality*.

* *Increased compared to basal (p < 0.05)*.

A *Increased compared to HPC1_HOTDOWN_*.

B *Increased from basal (main effect)*.

C *Increased compared to TPC1_TEMPFLAT_*.

### Metabolic and perceptual responses

Compared to basal, blood lactate concentrations (Table 3) increased following the hot downhill running trials (TPC2_HOTDOWN_; *F* = 11.0, *p* = 0.006, HPC1_HOTDOWN_; *F* = 13.3, *p* = 0.003 and HPC2_HOTDOWN_; *F* = 5.7, *p* = 0.035), but not the temperate flat trial (TPC1_TEMPFLAT_; *F* = 0.0, *p* = 0.874). Oxygen uptake ($\dot{V}$O_2_) increased (*F* = 236.0, *p* < 0.001) over time as a main effect but there was no difference between experimental trials (*P* < 0.05). Participants exercised at an average % $\dot{V}$O_2max_ of 70.2 ± 6.0% during the TPC1_TEMPFLAT_ trial, 70.8 ± 6.9% during the TPC2_HOTDOWN_ trial, 66.2 ± 6.0% during the HPC1_HOTDOWN_ trial and 65.8 ± 8.4% during the HPC2_HOTDOWN_ trial.

Both the rate of perceived exertion (RPE; Table 3) and thermal sensation (TS; Table 3) were greater during the hot downhill running trials (TPC2_HOTDOWN_ and HPC1_HOTDOWN_; *p* < 0.05) compared to the temperate flat trial (TPC1_TEMPFLAT_). No difference in RPE or TS was observed between the HPC1_HOTDOWN_ and HPC2_HOTDOWN_ trials (*p* > 0.05).

### Cellular stress (Hsp mRNA) response

**Figure 5:**
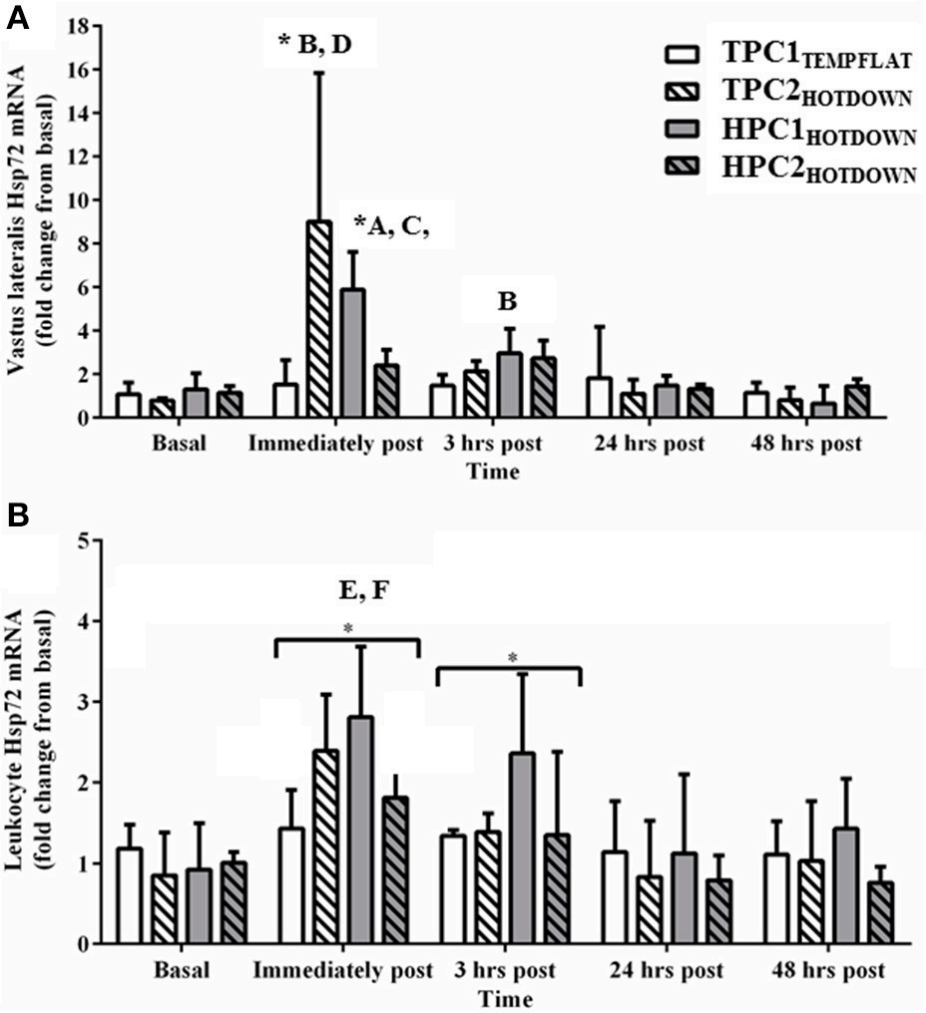
Hsp72 mRNA response immediately before, immediately post, 3 h post, 24 h post and 48 h post exercise in the Vastus lateralis **(A)** and Leukocytes **(B)**. ^*^ Increased (*p* < 0.001) compared to basal. A, increased (*p* = 0.020) in HPC1_HOTDOWN_ compared to TPC1_TEMPFLAT_. B, increased (*p* < 0.001) in TPC2_HOTDOWN_ compared to TPC1_TEMPFLAT_. C, increased (*p* = 0.028) in HPC1_HOTDOWN_ compared to HPC2_HOTDOWN_. D, increased in TPC2_HOTDOWN_ compared to HPC2_HOTDOWN_. E, HPC1_HOTDOWN_ increased (*p* = 0.049) compared to HPC2_HOTDOWN_. F, HPC1_HOTDOWN_ increased (*p* = 0.003) compared to TPC1_TEMPFLAT_. Data presented as median ± interquartile range.

The responses of Hsp72, Hsp90α, and Grp78 mRNA were assessed to determine their suitability as markers of the cellular stress response. Vastus lateralis Hsp72 mRNA (Figure 5A) increased as a main effect immediately post (*p* < 0.001) and 3 h post exercise (*p* = 0.002) compared to basal. Vastus lateralis Hsp72 mRNA increased immediately post exercise compared to basal in the hot downhill running trials (TPC2_HOTDOWN_ and HPC1_HOTDOWN_; *p* < 0.001). Vastus lateralis Hsp72 mRNA expression was greater immediately post TPC2_HOTDOWN_ and HPC1_HOTDOWN_ compared to the temperate flat trial (TPC1_TEMPFLAT_; *F* = 24.2, *p* < 0.001 and *F* = 9.2, *p* = 0.004, respectively) and the second hot downhill trial in the hot preconditioning group (HPC2_HOTDOWN_; *F* = 9.7, *p* = 0.003 and *F* = 5.0, *p* = 0.028, respectively). Vastus lateralis Hsp72 mRNA was also greater 3 h after HPC1_HOTDOWN_ compared to TPC1_TEMPFLAT_ (*F* = 6.6, *p* = 0.013).

Leukocyte Hsp72 mRNA expression (Figure 5B) increased as a main effect immediately post (*p* < 0.001) and 3 h post exercise (*p* = 0.004) compared to basal. Leukocyte Hsp72 mRNA expression was greater following HPC1_HOTDOWN_ compared to TPC1_TEMPFLAT_ (*F* = 4.2, *p* = 0.049) and HPC2_HOTDOWN_ (*F* = 10.2, *p* = 0.003).

**Figure 6:**
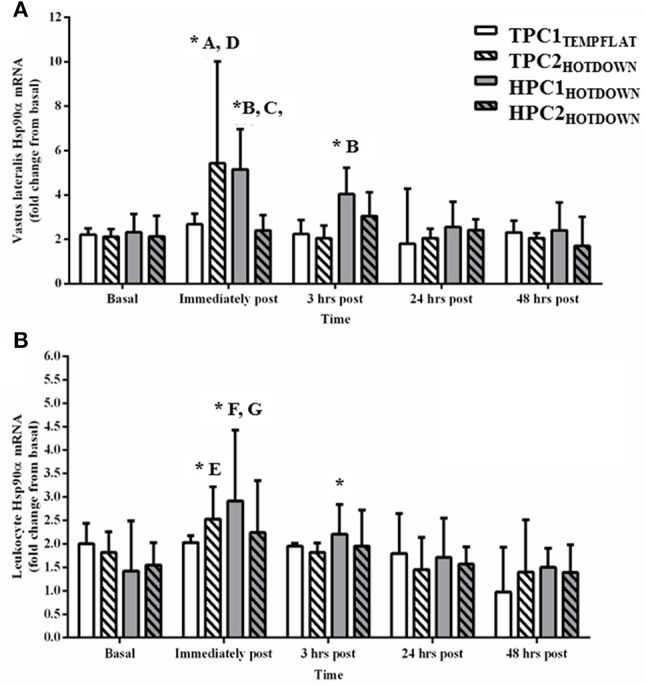
Hsp90α mRNA response immediately before, immediately post, 3 h post, 24 h post, and 48 h post exercise in the Vastus lateralis **(A)** and Leukocytes **(B)**. ^*^ Increased (*p* < 0.02) compared to basal. A, increased (*p* = 0.006) during TPC2_HOTDOWN_ compared to TPC1_TEMPFLAT_. B, increased (*p* < 0.001) during HPC1_HOTDOWN_ compared to HPC2_HOTDOWN_. C, increased (*p* < 0.05) during HPC1_HOTDOWN_ compared to TPC1_TEMPFLAT_. D, increased (*p* = 0.01) during TPC2_HOTDOWN_ compared to HPC2_HOTDOWN_. E, (*p* = 0.024) during TPC2_HOTDOWN_ compared to TPC1_TEMPFLAT_. F, increased (*p* = 0.030) during HPC1_HOTDOWN_ compared to HPC2_HOTDOWN_. G, (*p* = 0.002) during HPC1_HOTDOWN_ compared to TPC1_TEMPFLAT_. Data presented as median ± interquartile range.

Vastus lateralis Hsp90α mRNA (Figure 6A) increased compared to basal following the hot downhill running trials TPC2_HOTDOWN_ (immediately post exercise; *p* < 0.001) and HPC1_HOTDOWN_ (immediately post; *p* < 0.001 and 3 h post; *p* = 0.020). Vastus lateralis Hsp90α mRNA expression was greater immediately post TPC2_HOTDOWN_ compared to TPC1_TEMPFLAT_ (*F* = 8.4, *p* = 0.006), and HPC2_HOTDOWN_ (*F* = 7.4, *p* = 0.010). Vastus lateralis Hsp90α mRNA expression was also greater following HPC1_HOTDOWN_ compared to TPC1_TEMPFLAT_ (immediately post; *F* = 4.3, *p* = 0.044 and 3 h post; *F* = 4.4, *p* = 0.043) and HPC2_HOTDOWN_ (immediately post; *F* = 19.4, *p* < 0.001).

Leukocyte Hsp90α mRNA expression increased as a main effect immediately post exercise compared to basal (*p* < 0.001). Leukocyte Hsp90α mRNA expression also increased following TPC2_HOTDOWN_ (immediately post; *p* = 0.024) and HPC1_HOTDOWN_ (immediately post; *p* < 0.001 and 3 h post; *p* = 0.041) compared to basal. Leukocyte Hsp90α mRNA expression was greater immediately after TPC2_HOTDOWN_ compared to TPC1_TEMPFLAT_ (*F* = 5.3, *p* = 0.024). Leukocyte Hsp90α mRNA expression was also greater immediately after HPC1_HOTDOWN_ compared to TPC1_TEMPFLAT_ (*F* = 10.1, *p* = 0.002) and HPC2_HOTDOWN_ (*F* = 4.9, *p* = 0.030).

**Figure 7:**
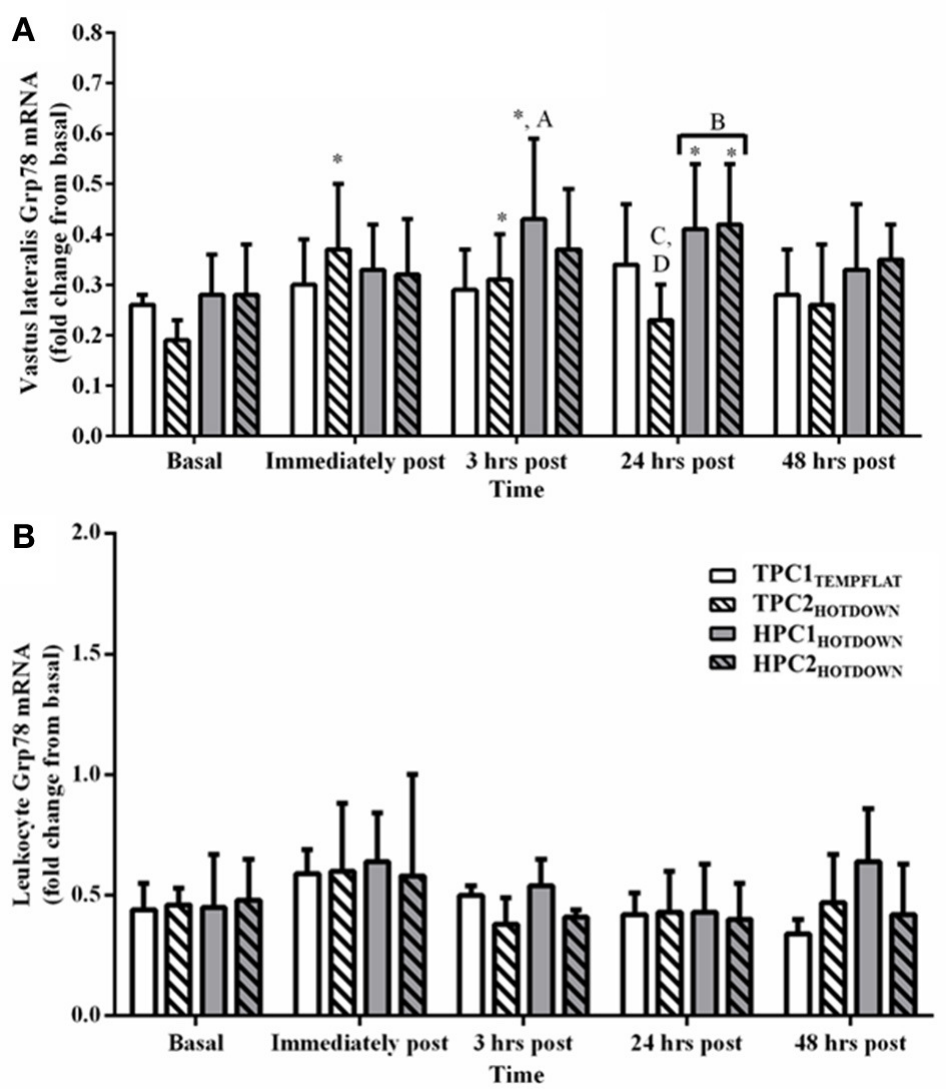
Grp78 mRNA response immediately before, immediately post, 3 h post, 24 h post, and 48 h post exercise in the Vastus lateralis **(A)** and Leukocytes **(B)**. ^*^ Increased (*p* < 0.01) compared to basal. ^*^ Increased (*p* < 0.01) compared to basal. A, increased (*p* = 0.031) during HPC1_HOTDOWN_ compared to TPC1_TEMPFLAT_. B, increased (*p* < 0.001) during HPC compared to TPC. C, decreased (*p* = 0.006) during TPC2_HOTDOWN_ compared to HPC2_HOTDOWN_. D, decreased (*p* = 0.01) during TPC2_HOTDOWN_ compared to TPC1_TEMPFLAT_. Data presented as median ± interquartile range.

Vastus lateralis Grp78 mRNA (Figure 7A) increased as a main effect immediately post to 48 h post exercise compared to basal (*p* < 0.002). Vastus lateralis Grp78 mRNA also increased within the TPC immediately post (*p* = 0.003) and within the HPC at 3 h (*p* < 0.001) and 24 h post (*p* < 0.001). Vastus lateralis Grp78 mRNA increased compared to basal following the hot downhill running trials, TPC2_HOTDOWN_ (immediately post; *p* < 0.001), HPC1_HOTDOWN_ (3 and 24 h post; *p* < 0.010) and HPC2_HOTDOWN_ (24 h post; *p* = 0.003), but did not change following the temperate flat trial (TPC1_TEMPFLAT_; *p* > 0.05).

All main effects and interactions had no effect (*p* > 0.05) on leukocyte Grp78 mRNA (Figure 7B).

### Relationship between mRNA responses

**Figure 8:**
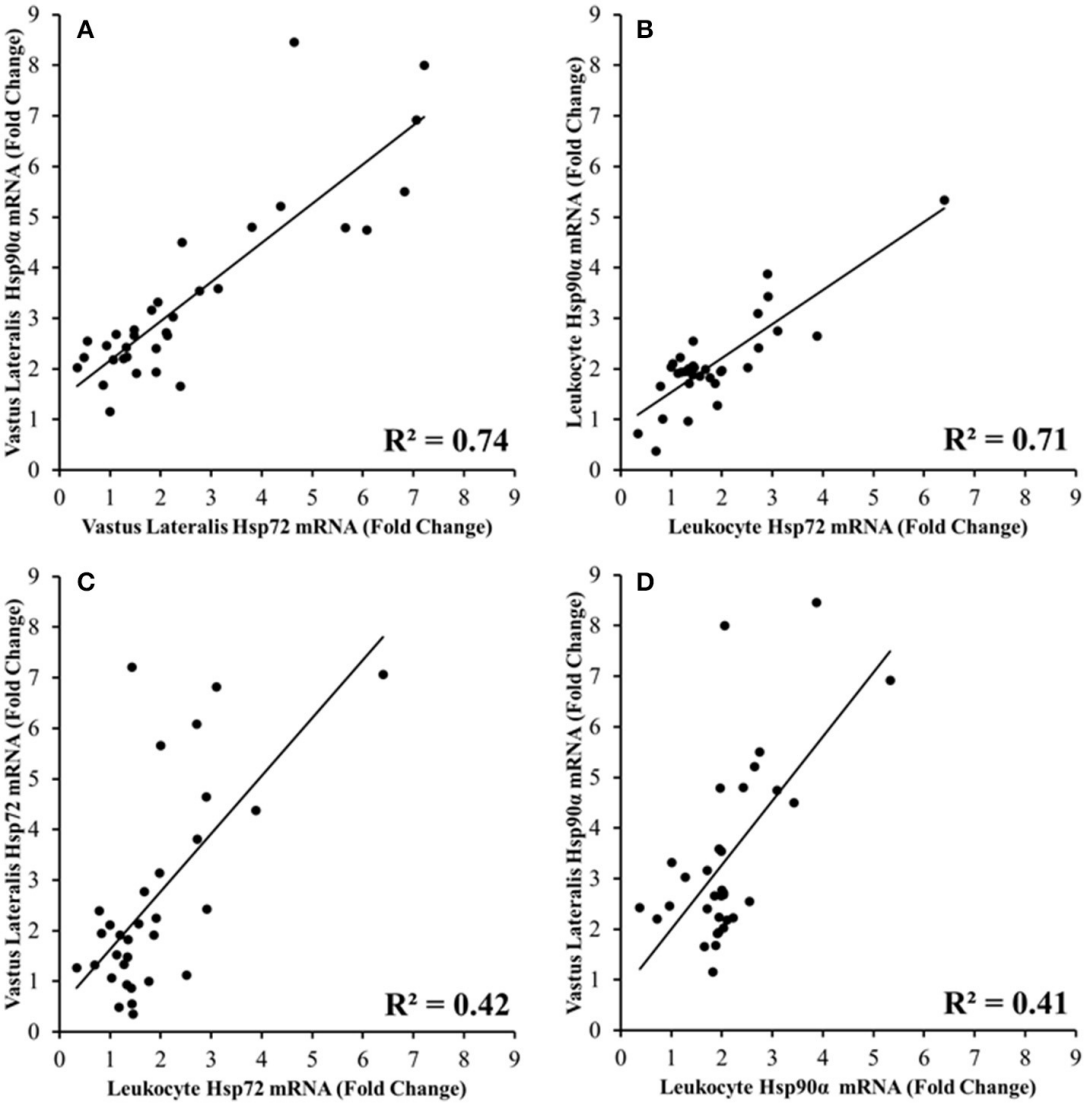
Relationships between Hsp72 **(A,C)** and Hsp90α mRNA **(B,D)** responses in the Vastus Lateralis and Leukocytes immediately before, immediately post, 3 h post TPC1_TEMPFLAT_ and HPC1_HOTDOWN_ (all *p* < 0.001).

A strong correlation was observed between vastus lateralis Hsp72 and Hsp90α mRNA expression (*r* = 0.863, *p* < 0.001; Figure 8A), and between leukocyte Hsp72 and Hsp90α mRNA expression (*r* = 0.844, *p* < 0.001; Figure 8B). Modest correlations were also observed between leukocyte Hsp72 mRNA and vastus lateralis Hsp72 mRNA (*r* = 0.651, *p* < 0.001; Figure 8C), and between leukocyte Hsp90α mRNA and vastus lateralis Hsp90α mRNA. (*r* = 0.640, *p* < 0.001; Figure 8D). Relationships between Hsp72 and Hsp90α mRNA, and Grp78 mRNA were not analyzed given the absence of a change in leukocyte Grp78 mRNA (Figure 7B).

A strong relationship was also observed between the peak T_re_ during TPC1_TEMPFLAT_ and HPC1_HOTDOWN_ and the immediately post measured leukocyte Hsp72 (*r* = 0.665, *p* = 0.026) and Hsp90α mRNA (*r* = 0.708, *p* = 0.015), and the 3 h measured leukocyte (*r* = 0.786, *p* = 0.004) and vastus lateralis (*r* = 0.720, *p* = 0.013) Hsp72 mRNA, and vastus lateralis Hsp90α mRNA (*r* = 0.682, *p* = 0.021). A strong relationship was also observed between peak heart rate during TPC1_TEMPFLAT_ and HPC1_HOTDOWN_, and leukocyte (*r* = 0.739, *p* = 0.009) and vastus lateralis (*r* = 0.766, *p* = 0.006) Hsp72 mRNA, and leukocyte (*r* = 0.677, *p* = 0.022) and vastus lateralis Hsp90α mRNA (*r* = 0.746, *p* = 0.008) at 3 h post exercise. No significant relationship was observed immediately post TPC1_TEMPFLAT_ or HPC1_HOTDOWN_.

## Discussion

The current study demonstrated that both VL and leukocyte Hsp72 and Hsp90α mRNA increases following the first trial of downhill running in a hot environment (HPC1_HOTDOWN_) were attenuated concurrently with reductions in exercising T_re_ and DOMS during the second trial of downhill running in a hot environment (HPC2_HOTDOWN;_ see Figures 5, 6). This suggests that the cellular stress response (Hsp72 and Hsp90α mRNA) occurred simultaneously within both tissues (Figure 8) and likely contributed to the preconditioning effect. This was not demonstrated in GRP78 mRNA (Figure 7). The absence of change in GRP78 mRNA in leukocytes suggests this is not an appropriate tissue to determine changes in its expression levels. Therefore, the leukocyte Hsp72 and Hsp90α mRNA responses could potentially be a useful surrogate for the VL response. At a physiological level the attenuated T_re_ (Figure 2), HR (Figure 3) and VAS (Figure 4) responses to an equivalent downhill run following HPC demonstrates an acute preconditioning response was attained. This was not discernible in the TPC group whom demonstrated the known responses to downhill running under heat stress in comparison to level gradient running in temperate conditions i.e., increased T_re_ (Figure 2), HR (Figure 3) and VAS (Figure 4).

### Cellular stress response and surrogate Hsp mRNA response

**Figure 9:**
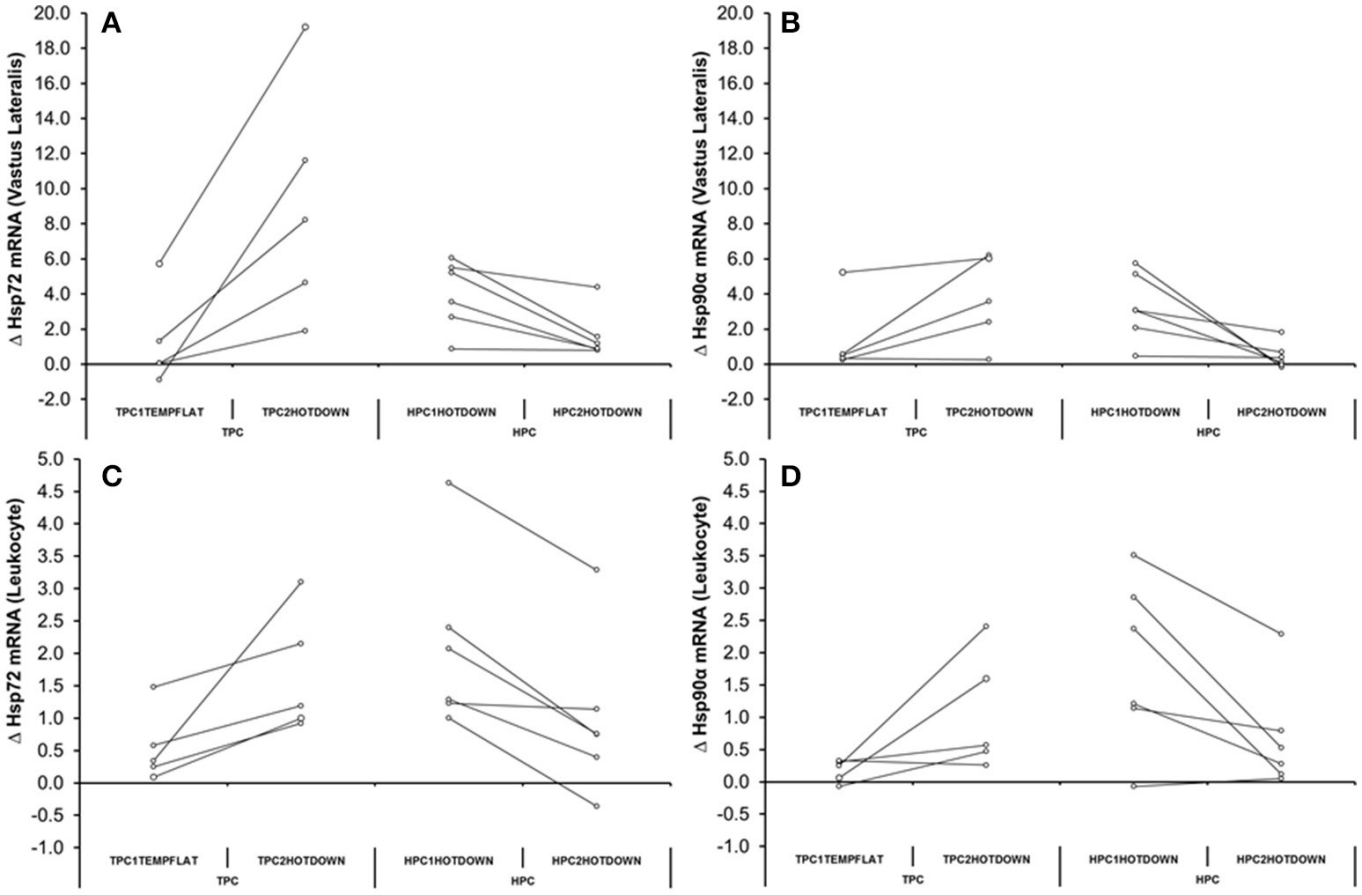
Individual responses reflecting the change in mRNA (**A** = Hsp72 mRNA in Vastus Lateralis, **B** = Hsp90α mRNA in Vastus Lateralis, **C** = Hsp72 mRNA in Leukocytes, D_S_ Hsp90α mRNA in Leukocytes) from baseline to immediately post TPC1_TEMPFLAT_ and TPC2_HOTDOWN_ **(A,C)**, and HPC1_HOTDOWN_ and HPC2_HOTDOWN_ **(B,D)**.

Increased Hsp72 mRNA transcription has frequently been demonstrated following exercise [leukocytes and VL (Walsh et al., 2001; Mestre-Alfaro et al., 2012)], muscle damaging exercise [VL (Vissing et al., 2009)] and exercise heat stress [leukocytes (Mestre-Alfaro et al., 2012)] within humans. However, there is less data available regarding the Hsp72 mRNA response being attenuated during repeated trials of muscle damaging exercise, or exercise heat stress as observed frequently during repeated trials of *in vitro* heat shock (Kiang et al., 1996; Theodorakis et al., 1999). Studies have previously only observed a blunted response following muscle damaging exercise in the VL (Paulsen et al., 2007) and exercise heat stress within leukocytes (Fehrenbach et al., 2001; Marshall et al., 2007). Within these studies reductions in thermal strain [exercising T_re_ (Fehrenbach et al., 2001; Marshall et al., 2007)] and muscle damage (Paulsen et al., 2007) during subsequent experimental trials were suggested to be responsible for the attenuated Hsp72 mRNA response observed. The current study also observed a reduction in thermal strain (T_re_ −0.3°C) equivalent to that of various heat acclimation regimes (Gibson et al., 2015b; Tyler et al., 2016), and an attenuated perceived muscle soreness (24 h post = +12.2%, 48 h post = −16.5%) response that is indicative of muscle damage (Fridén et al., 1981) from near identical exercise trials [HPC2_HOTDOWN_ compared to HPC1_HOTDOWN_ (see Figure 2 and Table 3)]. Together these responses indicate that downhill running models may be able to elicit a beneficial preconditioning effect (Dolci et al., 2015; Tuttle et al., 2015). Given that acute non-damaging exercise heat stress does not improve thermal responses to a greater extent than equivalent temperate condition exercise [Figure 2, (Lee et al., 2014)], the cellular responses to the eccentric muscle action of the damaging downhill running is important. The attenuated exercising T_re_ response could be suggestive of a reduction in relative exercise intensity and therefore potentially reduced requirement for ATP production (Febbraio et al., 1996), though no statistical difference in absolute intensity as indicated by $\dot{V}$O_2_ was observed (Table 3). Therefore, metabolic strain was likely reduced. Protein denaturation, the key cellular change associated with heat shock factor-1 (HSF-1) activation and Hsp72 and Hsp90α mRNA transcription, is temperature (Mestre-Alfaro et al., 2012), metabolic strain (Beckmann et al., 1992) and muscle damage (Michailidis et al., 2013) dependent. This suggests the observed attenuated thermal strain and muscle damage responses could be an important mechanism explaining the attenuated Hsp72 mRNA response in leukocytes (HPC1_HOTDOWN_ = +207%; HPC2_HOTDOWN_ = +79%) and VL (HPC1_HOTDOWN_ = +353%; HPC2_HOTDOWN_ = +109%) observed following the HPC2_HOTDOWN_ trial, compared to HPC2_HOTDOWN_ trial. Although, the expression of VL (Neubauer et al., 2014) and leukocyte (Moran et al., 2006) Hsp90α mRNA have previously been observed to increase following exercise and exercise heat stress, respectively, with equality of physiological stimuli i.e., T_re_ maintaining Hsp90α mRNA transcription (Gibson et al., 2015c), no studies have determined whether Hsp90α mRNA is attenuated during repeated trials of muscle damaging exercise. Consequently, the attenuated Hsp90α mRNA response in both leukocytes (HPC1_HOTDOWN_ = +106%, HPC2_HOTDOWN_ = +45%) and skeletal muscle (HPC1_HOTDOWN_ = +122%, HPC2_HOTDOWN_ = +113%) following reductions in physiological strain is a novel observation (see Figure 6). It is a novel finding that the relationship between Hsp72 and Hsp72 mRNA transcription is equivalent in the VL (Figure 8A, *R*^2^ = 0.74), as has been previously shown in leukocytes [*R*^2^ = 0.77 (Gibson et al., 2016)]. It has also been observed that the Hsp72 and Hsp72 mRNA transcription response is comparable following damaging exercise (Figure 8B, *R*^2^ = 0.71), as it has previously in non-damaging exercise models (Gibson et al., 2016). Within leukocytes, it has been observed that Hsp72 mRNA transcription (Gibson et al., 2015a,c; Mee et al., 2016), and Hsp90α mRNA transcription (Gibson et al., 2015c) returns to baseline 24 h following non-damaging exercise heat stress (Moran et al., 2006). The heat shock factor-1 (HSF-1) transcription pathway likely highlights the mechanism between equality of increases in Hsp72 and Hsp90α mRNA as demonstrated in this experiment (Figure 8), and others utilizing a non-damaging model (Gibson et al., 2016) with the attenuated mRNA response in the HPC2_HOTDOWN_ trial reflecting a reduction in the physiological stimuli as a result of the prior HPC for all participants (Figure 9).

HSP72 protein concentrations (due to translational inhibition) may not necessary directly represent the magnitude of the cellular stress response therefore the mRNA response has been proposed as more appropriate (Amorim et al., 2015; Gibson et al., 2015a; Lee et al., 2015). A reduction in the mRNA response is therefore representative of a gain in protein concentration (Marshall et al., 2007). The VL cellular adaptations associated with the repeated bout effect include a strengthened cytoskeleton [increased desmin concentrations (Feasson et al., 2002)] and elevated small HSP concentrations [αβ-crystallin and HSP27 (Paulsen et al., 2009)] and therefore, could be responsible for the attenuated Hsp72 and Hsp90α mRNA responses observed following HPC2_HOTDOWN_. Optimization of transcriptional and translational processes (Touchberry et al., 2012) and elevated concentrations of anti-apoptotic (Horowitz, 2014) and antioxidant (Horowitz and Kodesh, 2010) proteins, which are implicated in enhanced thermotolerance, could also be responsible for the attenuated Hsp72 and Hsp90α mRNA responses observed following HPC2_HOTDOWN_ within both the VL and leukocytes.

The current study observed for the first time that the leukocyte and VL Hsp72 and Hsp90α mRNA response occurs concurrently (Figures 8C,D). This novel data supports the notion that leukocytes are a desirable tissue site for determining the cellular stress response due to accessibility for analysis following exposure to both systemic signals and to signals of the perfused tissues (Sonna et al., 2007). Some caution should be raised as this experiment did not quantify the leukocyte infiltration to skeletal muscle, a known component of the intramuscular response which follows damaging exercise (Malm et al., 2004), though the time course and magnitude of this response are controversial (St. Pierre Schneider and Tiidus, 2007). A resolution to this issue within future experiments would be quantification of total mRNA (Sanders et al., 2014). As previously discussed the reduction in thermal and metabolic strain mediated within both leukocytes and the VL likely attenuated the increases in protein denaturation during HPC2_HOTDOWN_ and thus could explain the attenuated Hsp72 and Hsp90α mRNA response observed in both tissues. Muscle damage mediated release of ligands [damage associated molecular patterns (DAMPs), circulating cell free DNA and extracellular HSPs (Neubauer et al., 2014)] from skeletal muscle could also explain the concurrent Hsp72 and Hsp90α mRNA responses via a toll like receptor mediated stress response within leukocytes, as previously observed following muscle damaging exercise (Fernandez-Gonzalo et al., 2012). Although elevations in these ligands may be exercise related (Neubauer et al., 2013), evidence for these ligands actually being released from skeletal muscle following exercise is limited. Consequently, the concurrent Hsp72 and Hsp90α mRNA responses are probably dependent on increases in thermal strain and metabolic strain within both leukocytes and the VL, and are unlikely to be muscle damage dependent.

Increases in VL Grp78 mRNA were observed following both HPC1_HOTDOWN_ and HPC2_HOTDOWN_ despite the observed reductions in exercising T_re_ and DOMS, which are associated with reduced protein denaturation, the key cellular change regulating Grp78 mRNA transcription. Activation of the unfolded protein response also occurs when the endoplasmic reticulum protein load increases during cellular remodeling (Ron and Walter, 2007). Therefore, the Grp78 mRNA response may reflect the need to increase ER protein folding capacity to aid cellular adaptation (Ron and Walter, 2007). These observations combined with the absence of Grp78 mRNA increases within leukocytes suggest that Grp78 mRNA cannot be used as a marker of the cellular stress response, or thermotolerance, at least within the current experimental model.

### Practical applications and future directions

The results of this experiment highlight that an acute bout of downhill running in a hot environment is an effective preconditioning strategy to attenuate the increase in thermal strain experienced during a subsequent, equivalent exercise in hot conditions. Typically it is proposed that athletes, workers and the military should perform acclimation/acclimatization prior to traveling to unfamiliar, hot conditions (Racinais et al., 2015). An acute bout of downhill running in hot conditions i.e., whole body preconditioning may therefore be there an appropriate method to expediently elicit thermal protection i.e., a reduction in thermal strain prior to exercise in hot conditions. Given recent evidence of cross acclimation between stressors (Gibson et al., 2015c; Lee et al., 2016; White et al., 2016), it is also possible that this whole body preconditioning strategy will induce physiological and cellular adaptations which are beneficial in unfamiliar stressors e.g., hypoxia. These adaptations may become greater with repeated stress, i.e., repeated HPC, thus providing either a greater magnitude of cytoprotection, or a more prolonged post-HPC level of protection, or a combination of both. It is currently unknown how long the preconditioning effect elicited by HPC1_HOTDOWN_ is retained beyond the 7 d duration we have observed. Without evidencing the decay in HSP72 and HSP90α content this is difficult to estimate, as such this remains an area for future investigation. Measurement of RNA/protein ratios may also aid understanding of the cytoprotective dynamics. The current study suggests that the leukocyte Hsp72 and Hsp90α mRNA responses could potentially be used as a surrogate measure of the HSR within skeletal muscle, at least within the current experimental model (preconditioning via downhill running in a hot environment). Consequently, the leukocyte Hsp72 and Hsp90α mRNA responses are potentially a relevant marker of individuals thermotolerance and thus could be useful for allocating appropriate athletic or occupational workloads without the potential reductions in performance and increased infection risk (within the biopsy incision) associated with skeletal muscle biopsies. The current experimental model utilized a combination of exercise heat stress and downhill running. Consequently, leukocyte Hsp72 mRNA and Hsp90α mRNA responses could be useful for suggesting thermotolerance within situations where exercise heat stress occur, such as military exercises or during athletic competition. Although the concurrent Hsp72 and Hsp90α mRNA responses are unlikely to be mechanistically linked exclusively to a muscle damage response, future work should set out to confirm whether this concurrent leukocyte and skeletal muscle response also occurs within a non-damaging exercise heat stress trial.

### Summary and conclusions

Hot downhill running is an effective preconditioning strategy which ameliorates physiological strain, muscle soreness and the cellular stress response (Hsp72 and Hsp90α mRNA transcription) to a subsequent bout of exercise-heat stress. This preconditioning strategy has applications for athletic, occupational and military populations. The current study suggests that Hsp72 and Hsp90α mRNA act as markers of the cellular stress response within both the VL and leukocytes. Consequently, the leukocyte Hsp72 mRNA and Hsp90α mRNA responses appear to be a surrogate measure of the cellular stress response in the VL. Accordingly, venepuncture to obtain circulating leukocytes provides a viable alternative to muscle sampling via biopsies to determine the cellular stress response to exercise-heat stress.

## Author contributions

JT, PC, LT, and ML conception and design of research; JT, JB, DH, AJM, OP, CK, FR, and SA performed experiments; JT, BC, OG, PC, AWM, LT, and ML analyzed data; JT, BC, OG, PC, LT, and ML interpreted results of experiments; JT and OG prepared figures; JT drafted manuscript; JT, BC, OG, JB. DH, AJM, OP, CK, FR, SA, PC, AJM, AWM, LT, and ML edited and revised manuscript; JT, BC, OG, JB, DH, AJM, OP, CK, FR, SA, PC, AJM, AWM, LT, and ML approved the final version of manuscript.

### Conflict of interest statement

The authors declare that the research was conducted in the absence of any commercial or financial relationships that could be construed as a potential conflict of interest.

## Abbreviations

CT: cycling threshold
DOMS: Delayed onset muscle soreness
Grp78: Glucose regulated protein 78
HOT: Hot testing conditions
HPC: Hot preconditioning group
Hsp: Heat shock protein (number indicates molecular weight)
HSF-1: Heat Shock factor-1
HSR: Heat shock response
LT: Lactate threshold
mRNA: Messenger RNA
PBS: Phosphate-buffered saline
QT: Quadriceps tenderness
RH: Relative humidity
RNA: Ribonucleic acid
RPE: Rating of perceived exertion
RT-QPCR: Reverse transcription quantitative polymerase chain reaction
TEMP: Temperate testing conditions
TPC: Temperate preconditioning group
TS: Thermal sensation
UOsm: Urine Osmolality
VL: Vastus lateralis
$\dot{V}$O_2_: Oxygen uptake
$\dot{V}$O_2max_: Maximal oxygen uptake.
